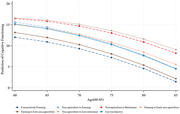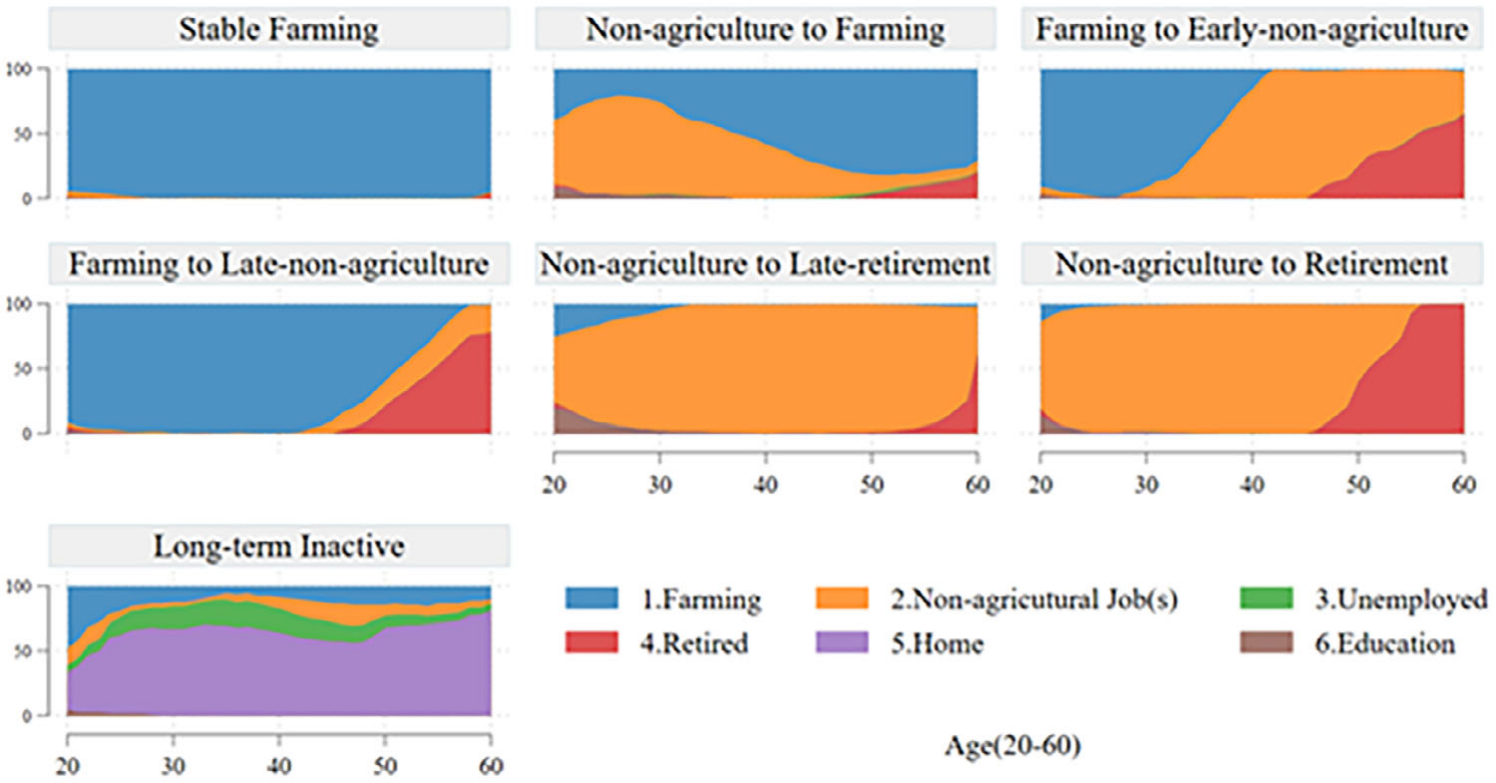# Trajectories of work‐history and their associations with cognitive impairment in later life

**DOI:** 10.1002/alz.090040

**Published:** 2025-01-09

**Authors:** Peipei Fu, Yi Wang

**Affiliations:** ^1^ Shandong University, Jinan, Shandong China; ^2^ Yale University, New Heaven, CT USA

## Abstract

**Background:**

The associations between work‐history trajectories and cognitive impairment among older adults were unknown. We investigated the association between work‐history patterns and cognitive trajectories in later life.

**Method:**

We conducted the study by using four regular waves and Life History survey in the Harmonized Dataset of the China Health and Retirement Longitudinal Study (CHARLS), included 5511 participants (47.5% were female, 2619/5511) aged 60 years or older in China. Cognitive function was measured using Telephone Interview for Cognitive Status (TICS). We utilized sequence analysis to model the work‐history patterns of each individual between age 20 and 60. All work sequences are grouped into 7 trajectories, distinguished by salient occupational characteristics and dynamic career changes over the entire observation period. Growth curve modeling was performed to capture how the life course of work impacts both the static level and dynamic trend of the elderly’s cognitive function.

**Result:**

Respondents with Stable Farming pattern displayed a 1.73 lower average cognitive functioning scores (p<0.001), and respondents belong to Farming to Late‐Non‐Agriculture had a 1.12 lower level (p<0.05). Respondents with Non‐agriculture to Late‐retirement or long‐run inactive patterns initially started with higher levels of cognitive functioning and declined at lower rates. Conversely, respondents with stable farming work pattern exhibited the worst cognitive functioning, in terms of both initial level and decline rate. No apparent evidence suggested there are significant differences on cognitive faculty in later life between the reference group and cluster 2 (Non‐Agriculture to Farming), cluster 5 (Non‐Agriculture to Late‐Retirement), cluster 3(Long‐term Inactive) and cluster 7(Long‐term Inactive).

**Conclusion:**

We identified people who remained in farming enduringly or for prolonged durations showed diminished cognitive functioning levels and a heightened susceptibility to cognitive decline. Hence, it is of paramount importance to direct attention towards the cognitive well‐being of farmers or individuals with a substantial history of farming as they progress into their later years.